# 2-Chloro­pyrimidin-4-amine

**DOI:** 10.1107/S1600536811055863

**Published:** 2012-01-07

**Authors:** Gerard A. van Albada, Mohamed Ghazzali, Khalid Al-Farhan, Jan Reedijk

**Affiliations:** a Leiden Institute of Chemistry, Leiden University, PO Box 9502, 2300 RA Leiden, The Netherlands; b Department of Chemistry, Faculty of Science, King Saud University, PO Box 2455, Riyadh 11451, Saudi Arabia

## Abstract

In the title pyrimidine derivative, C_4_H_4_ClN_3_, the 2-chloro and 4-amino substituents almost lie in the mean plane of the pyrimidine ring, with deviations of 0.003 (1) Å for the Cl atom, and 0.020 (1) Å for the N atom. In the crystal, molecules are linked *via* pairs of N—H⋯N hydrogen bonds, forming inversion dimers. These dimers are further linked *via* N—H⋯N hydrogen bonds, forming an undulating two-dimensional network lying parallel to (100).

## Related literature

For compounds related to pyrimidin-4-amine, see: Van Albada *et al.* (1999 ▶, 2003 ▶); Van Meervelt & Uytterhoeven (2003 ▶); Kožíšek *et al.* (2005 ▶). For the agricultural and pharmaceutical relevance of 2-chloro­pyrimidin-4-amine, see: Zunszain *et al.* (2005 ▶). For graph-set analysis of hydrogen bonds, see: Etter *et al.* (1990 ▶); Bernstein *et al.* (1995 ▶).
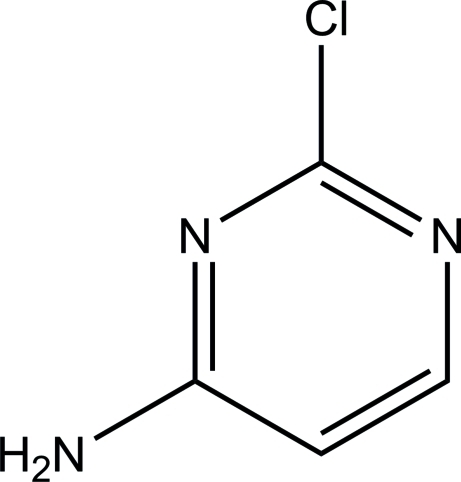


## Experimental

### 

#### Crystal data

C_4_H_4_ClN_3_
*M* *_r_* = 129.55
Monoclinic, 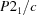

*a* = 3.83162 (19) Å
*b* = 11.8651 (7) Å
*c* = 12.7608 (7) Å
β = 100.886 (2)°
*V* = 569.70 (5) Å^3^
*Z* = 4
Mo *K*α radiation
μ = 0.55 mm^−1^
*T* = 294 K
0.40 × 0.20 × 0.20 mm

#### Data collection

Rigaku R-AXIS RAPID diffractometer
Absorption correction: multi-scan (*CrystalClear*; Rigaku, 2007 ▶) *T*_min_ = 0.840, *T*_max_ = 0.888
9506 measured reflections
1296 independent reflections
962 reflections with *I* > 2σ(*I*)
*R*_int_ = 0.038

#### Refinement

*R*[*F* ^2^ > 2σ(*F* ^2^)] = 0.035
*wR*(*F* ^2^) = 0.092
*S* = 1.14
1296 reflections
82 parameters
2 restraints
H atoms treated by a mixture of independent and constrained refinement
Δρ_max_ = 0.17 e Å^−3^
Δρ_min_ = −0.27 e Å^−3^

### 

Data collection: *CrystalClear* (Rigaku, 2007 ▶); cell refinement: *CrystalClear*; data reduction: *CrystalClear*; program(s) used to solve structure: *SHELXS97* (Sheldrick, 2008 ▶); program(s) used to refine structure: *SHELXL97* (Sheldrick, 2008 ▶); molecular graphics: *DIAMOND* (Brandenburg, 2007 ▶); software used to prepare material for publication: *publCIF* (Westrip, 2010 ▶).

## Supplementary Material

Crystal structure: contains datablock(s) I, global. DOI: 10.1107/S1600536811055863/zj2047sup1.cif

Structure factors: contains datablock(s) I. DOI: 10.1107/S1600536811055863/zj2047Isup2.hkl

Supplementary material file. DOI: 10.1107/S1600536811055863/zj2047Isup3.cml

Additional supplementary materials:  crystallographic information; 3D view; checkCIF report

## Figures and Tables

**Table 1:** Hydrogen-bond geometry (Å, °)

*D*—H⋯*A*	*D*—H	H⋯*A*	*D*⋯*A*	*D*—H⋯*A*
N2—H2*A*⋯N3^i^	0.90 (2)	2.17 (2)	3.069 (2)	174 (2)
N2—H2*B*⋯N1^ii^	0.87 (2)	2.16 (2)	3.024 (2)	170 (2)

Symmetry codes: (i) 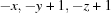; (ii) 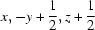.

**Fig. 1.**
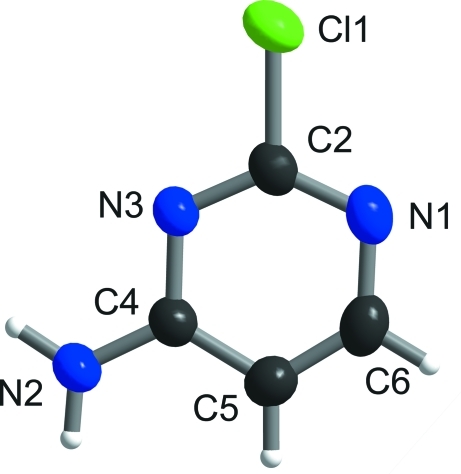
Atomic numbering scheme and thermal ellipsoidal (50% probability level) of the title compound. Hydrogen atoms are presented as spheres of arbitrary radii.

**Fig. 2.**
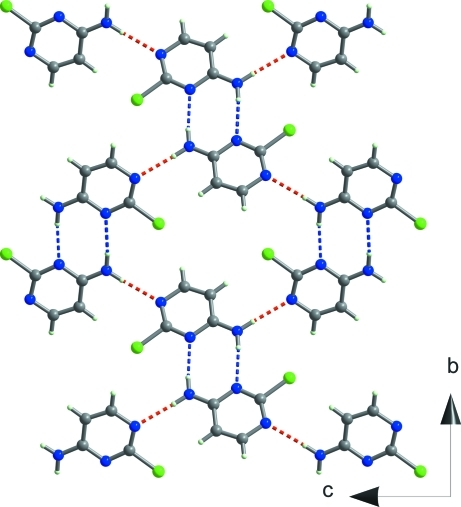
bc-plane projection showing the N—H···N hydrogen bonds as dotted line of R22(8) loop (presented in blue color), and C(5) chain (presented in red color). Symmetry codes: (i) -x, -y + 1, -z + 1; (ii) x, -y + 1/2, z + 1/2.

## supplementary crystallographic information

### Comment

The molecule of 2-chloropyrimidin-4-amine is relevant for agrochemistry as a plant growth regulator and as a pharmaceutical intermediate (Zunszain *et al.* 2005). It could also be an interesting precursor for chelating ligands after chlorine substitution. Pyrimidin-amines are interesting bridging ligands, as they contain two nitrogen coordination donor atoms, and an amine as a hydrogen bond donor group (Van Albada *et al.* 1999, 2003). The ligands pyrimidin-4-amine and 2-amine can easily bridge two metal ions (Kožíšek *et al.* 2005). With the presence of two donor atoms, the title compound might serve as a building block in the formation of coordination polymers. Due to the position of a chloride atom in-between the two donor N atoms of the pyrimidin-4-amine, the bridging would be likely to change. In fact, coordination complexes with the 2-chloropyrimidin-4-amine are yet unreachable. We here present the molecular structure of this compound, (Figure 1).

The 2-chloropyrimidin-4-amine molecule is nearly planar, with r.m.s. deviation of the pyrimidine heterocyclic non-hydrogen atoms is 0.002 (2) Å. In the crystal, molecules are arranged with two N—H···N hydrogen bond motifs, where the amine group serves as a twofold donor of the hydrogen atoms for the two pyrimidine nitrogen atoms. Considering graph-set analysis (Etter *et al.*, 1990; Bernstein *et al.*, 1995), the descriptors are *R*^2^_2_(8) loops and C(5) chain motifs along the [001] and [010] vectors, respectively. The network can be described as a wobbled two-dimensional network extending in the (100) plane, (Figure 2). It is worth to note that the related pyrimidin-4-amine molecule (Van Meervelt *et al.* 2003), crystallizes in the orthorhombic *Pcab* space group and exhibits only the N—H···N hydrogen bond with C(5) chain motif of a one-dimensional *zigzag* chain.

### Experimental

The ligand was used as commercially available. 0.5 mg of the compound was dissolved in 10 ml of methanol. The solution was stand at room temperature in a closed vessel. After two weeks, colourless blocks appeared and separated by filtration.

### Refinement

Carbon-bound H-atoms were placed in ideal calculated positions [aromatic C—H 0.93 Å, *U*_iso_(H) = 1.2*U*_eq_(C)] and refined as riding atoms. The amine H-atoms were constrained into their positions using two distance restraints [N—H 0.91 Å, *U*_iso_(H) = 1.2*U*_eq_(N)].

### Crystal data

**Table:**

C_4_H_4_ClN_3_	*F*(000) = 264
*M**_r_* = 129.55	*D*_x_ = 1.510 Mg m^−^^3^
Monoclinic, *P*2_1_/*c*	Mo *K*α radiation, λ = 0.71075 Å
Hall symbol: -P 2ybc	Cell parameters from 342 reflections
*a* = 3.83162 (19) Å	θ = 3.3–27.5°
*b* = 11.8651 (7) Å	µ = 0.55 mm^−^^1^
*c* = 12.7608 (7) Å	*T* = 294 K
β = 100.886 (2)°	Block, colourless
*V* = 569.70 (5) Å^3^	0.40 × 0.20 × 0.20 mm
*Z* = 4	

### Data collection

**Table:**

Rigaku R-AXIS RAPID diffractometer	1296 independent reflections
Radiation source: fine-focus sealed tube	962 reflections with *I* > 2σ(*I*)
graphite	*R*_int_ = 0.038
ω scans	θ_max_ = 27.5°, θ_min_ = 3.3°
Absorption correction: multi-scan (*CrystalClear*; Rigaku, 2007)	*h* = −4→4
*T*_min_ = 0.840, *T*_max_ = 0.888	*k* = −15→15
9506 measured reflections	*l* = −16→16

### Refinement

**Table:**

Refinement on *F*^2^	Primary atom site location: structure-invariant direct methods
Least-squares matrix: full	Secondary atom site location: difference Fourier map
*R*[*F*^2^ > 2σ(*F*^2^)] = 0.035	Hydrogen site location: inferred from neighbouring sites
*wR*(*F*^2^) = 0.092	H atoms treated by a mixture of independent and constrained refinement
*S* = 1.14	*w* = 1/[σ^2^(*F*_o_^2^) + (0.0422*P*)^2^ + 0.0697*P*] where *P* = (*F*_o_^2^ + 2*F*_c_^2^)/3
1296 reflections	(Δ/σ)_max_ < 0.001
82 parameters	Δρ_max_ = 0.17 e Å^−^^3^
2 restraints	Δρ_min_ = −0.27 e Å^−^^3^

### Special details

**Table:**

Geometry. All e.s.d.'s (except the e.s.d. in the dihedral angle between two l.s. planes) are estimated using the full covariance matrix. The cell e.s.d.'s are taken into account individually in the estimation of e.s.d.'s in distances, angles and torsion angles; correlations between e.s.d.'s in cell parameters are only used when they are defined by crystal symmetry. An approximate (isotropic) treatment of cell e.s.d.'s is used for estimating e.s.d.'s involving l.s. planes.
Refinement. Refinement of *F*^2^ against ALL reflections. The weighted *R*-factor *wR* and goodness of fit *S* are based on *F*^2^, conventional *R*-factors *R* are based on *F*, with *F* set to zero for negative *F*^2^. The threshold expression of *F*^2^ > σ(*F*^2^) is used only for calculating *R*-factors(gt) *etc*. and is not relevant to the choice of reflections for refinement. *R*-factors based on *F*^2^ are statistically about twice as large as those based on *F*, and *R*- factors based on ALL data will be even larger.

### Fractional atomic coordinates and isotropic or equivalent isotropic displacement parameters (Å^2^)

**Table:**

	*x*	*y*	*z*	*U*_iso_*/*U*_eq_	
Cl1	0.05814 (13)	0.43867 (4)	0.20898 (3)	0.0586 (2)	
N1	0.3425 (4)	0.25622 (13)	0.29987 (11)	0.0500 (4)	
N2	0.2112 (5)	0.37262 (14)	0.59166 (12)	0.0522 (4)	
H2B	0.277 (5)	0.3340 (17)	0.6504 (14)	0.065 (6)*	
H2A	0.103 (5)	0.4395 (14)	0.5959 (17)	0.061 (6)*	
C2	0.2035 (4)	0.35294 (14)	0.32044 (13)	0.0419 (4)	
N3	0.1530 (4)	0.39673 (11)	0.41103 (10)	0.0407 (3)	
C4	0.2612 (4)	0.33227 (13)	0.49910 (12)	0.0400 (4)	
C5	0.4177 (5)	0.22616 (15)	0.48826 (14)	0.0480 (4)	
H5	0.4961	0.1806	0.5473	0.058*	
C6	0.4495 (5)	0.19310 (16)	0.38937 (16)	0.0531 (5)	
H6	0.5506	0.1230	0.3818	0.064*	

### Atomic displacement parameters (Å^2^)

**Table:**

	*U*^11^	*U*^22^	*U*^33^	*U*^12^	*U*^13^	*U*^23^
Cl1	0.0718 (4)	0.0665 (4)	0.0379 (3)	0.0003 (2)	0.0113 (2)	0.0060 (2)
N1	0.0570 (9)	0.0502 (9)	0.0447 (9)	0.0005 (7)	0.0143 (7)	−0.0103 (7)
N2	0.0775 (11)	0.0455 (9)	0.0345 (8)	0.0079 (8)	0.0130 (7)	0.0007 (7)
C2	0.0431 (9)	0.0456 (9)	0.0379 (9)	−0.0055 (7)	0.0101 (7)	−0.0045 (7)
N3	0.0496 (8)	0.0380 (7)	0.0357 (7)	−0.0010 (6)	0.0112 (6)	−0.0019 (6)
C4	0.0439 (9)	0.0395 (9)	0.0372 (8)	−0.0034 (7)	0.0092 (7)	−0.0013 (7)
C5	0.0533 (10)	0.0429 (10)	0.0472 (10)	0.0047 (8)	0.0075 (8)	0.0027 (8)
C6	0.0550 (11)	0.0442 (10)	0.0610 (12)	0.0047 (8)	0.0132 (9)	−0.0092 (9)

### Geometric parameters (Å, °)

**Table:**

Cl1—C2	1.7518 (17)	C2—N3	1.315 (2)
N1—C2	1.312 (2)	N3—C4	1.358 (2)
N1—C6	1.363 (2)	C4—C5	1.412 (2)
N2—C4	1.322 (2)	C5—C6	1.349 (2)
N2—H2B	0.874 (15)	C5—H5	0.9300
N2—H2A	0.902 (16)	C6—H6	0.9300
			
C2—N1—C6	112.47 (15)	N2—C4—C5	123.11 (16)
C4—N2—H2B	120.6 (14)	N3—C4—C5	119.33 (15)
C4—N2—H2A	121.3 (14)	C6—C5—C4	117.77 (16)
H2B—N2—H2A	118 (2)	C6—C5—H5	121.1
N1—C2—N3	130.85 (16)	C4—C5—H5	121.1
N1—C2—Cl1	115.10 (12)	C5—C6—N1	123.94 (17)
N3—C2—Cl1	114.05 (13)	C5—C6—H6	118.0
C2—N3—C4	115.64 (14)	N1—C6—H6	118.0
N2—C4—N3	117.56 (15)		

### Hydrogen-bond geometry (Å, °)

**Table:**

*D*—H···*A*	*D*—H	H···*A*	*D*···*A*	*D*—H···*A*
N2—H2A···N3^i^	0.90 (2)	2.17 (2)	3.069 (2)	174 (2)
N2—H2B···N1^ii^	0.87 (2)	2.16 (2)	3.024 (2)	170 (2)

Symmetry codes: (i) −*x*, −*y*+1, −*z*+1; (ii) *x*, −*y*+1/2, *z*+1/2.
